# Feature Extraction of Upper Airway Dynamics during Sleep Apnea using Electrical Impedance Tomography

**DOI:** 10.1038/s41598-020-58450-4

**Published:** 2020-01-31

**Authors:** Ghazal Ayoub, Thi Hang Dang, Tong In Oh, Sang-Wook Kim, Eung Je Woo

**Affiliations:** 1 Department of Biomedical Engineering, Graduate School, Kyung Hee University, Yongin, Korea; 2 Department of Medical Engineering, Graduate School, Kyung Hee University, Seoul, Korea; 3 Department of Otorhinolaryngology, Gyeongsang National University College of Medicine and Gyeongsang National University Hospital, Jinju, Korea

**Keywords:** Respiratory distress syndrome, Translational research

## Abstract

Characterizing upper airway occlusion during natural sleep could be instrumental for studying the dynamics of sleep apnea and designing an individualized treatment plan. In recent years, obstructive sleep apnea (OSA) phenotyping has gained attention to classify OSA patients into relevant therapeutic categories. Electrical impedance tomography (EIT) has been lately suggested as a technique for noninvasive continuous monitoring of the upper airway during natural sleep. In this paper, we developed the automatic data processing and feature extract methods to handle acquired EIT data for several hours. Removing ventilation and blood flow artifacts, EIT images were reconstructed to visualize how the upper airway collapsed and reopened during the respiratory event. From the time series of reconstructed EIT images, we extracted the upper airway closure signal providing quantitative information about how much the upper airway was closed during collapse and reopening. Features of the upper airway dynamics were defined from the extracted upper airway closure signal and statistical analyses of ten OSA patients’ data were conducted. The results showed the feasibility of the new method to describe the upper airway dynamics during sleep apnea, which could be a new step towards OSA phenotyping and treatment planning.

## Introduction

Sleep apnea is a sleep disorder with an increasing prevalence especially in obese population. Untreated sleep apnea may increase the risk of hypertension, stroke, diabetes, high blood pressure, and other ailments^1^. There are three types of sleep apnea: obstructive, central and mixed. Obstructive sleep apnea (OSA) refers to the blockage of the upper airway for longer than ten seconds and central sleep apnea (CSA) refers to the cessation of airflow without any respiratory effort. Mixed apnea is a combination of obstructive and central sleep apnea symptoms^2^. There are four major treatment methods for sleep apnea including positive airway pressure (PAP), surgery, oral appliance and upper airway stimulation (UAS)^3^. Currently, PAP is the primary treatment modality for patients with moderate or severe OSA^4^. PAP therapy has been shown to significantly decrease the apnea hypopnea index (AHI) that is the average number of apnea and hypopnea events during one hour of sleep at night. However, PAP adherence has not currently reached an acceptable level since about 50% discontinue PAP therapy within the first year^5^. Though several surgical methods can be adopted for about 5% of OSA patients, their outcomes remain relatively poor in some patients^6^. The oral device is usually recommended for mild or moderate OSA patients who could not tolerate the PAP treatment. Requiring surgery for device implantation, the UAS has been recently used only for properly selelcted patients, but its effectiveness varies among the patients. OSA treatments, therefore, could be costly, time-consuming and discouraging.

Considering that most OSA patients remain untreated or undiagnosed^7,8^, understanding various underlying causes of sleep apnea is clearly needed to design patient-specific and tolerable treatment methods^9,10^. OSA phenotyping is a term frequently used in the literature to categorize OSA patients by a pathognomonic characteristic or combination of disease features related to clinically meaningful attributes^11,12^. Noting that features related to the upper airway dynamics during sleep apnea can be potentially useful for OSA phenotyping, real-time imaging of the upper airway during natural sleep was lately proposed using the electrical impedance tomography (EIT) technique^13,14^. Feasibility of the new EIT imaging method was demonstrated to estimate the size and shape of the upper airway during OSA events.

In this paper, we introduce an automatic EIT data processing method to extract a set of features to describe the upper airway dynamics related to sleep apnea. After explaining the details of the automatic feature extraction method, results of applying it to the acquired polysomnography (PSG) and EIT data from ten OSA patients are presented. Upper airway dynamics of all ten patients are compared using the extracted features including the maximum upper airway closure, duration, mean UA % closure, slew rate, fall rate, rise/fall time ratio, plateau percent, and fluctuation. The upper airway dynamics are also compared between two different respiratory events of obstructive apnea and hypopnea.

## Materials and Methods

### PSG and EIT data collections from OSA patients

Ten patients with moderate and severe OSA were recruited for this study. At the beginning of each study, the patient was requested to employ a swallowing maneuver while collecting EIT data using a custom designed EIT device (KHU Mark2.5, IIRC, Korea)^15,16^. EIT images of the closed upper airway were reconstructed as time-difference images between normal breathing and swallowing. In our previous study^14^, these EIT images were compared with MRI images of the upper airway to confirm that the EIT images correctly visualized the closed upper airway. The EIT images of the upper airway will be utilized in the automatic data processing process described in the next section.

During patients’ natural sleep, PSG and EIT data were simultaneously acquired using a PSG device (Somte, Compumedics, Melbourne, Australia) and the EIT device (KHU Mark2.5, IIRC, Korea)^15,16^. Sleep apnea was manually scored according to the guideline of the American Academy of Sleep Medicine (AASM) using the PSG data^17^. Eight patients had obstructive apneic events, seven had hypopneic events, two had mixed events, and only one had central apnea events. The synchronized EIT and PSG data were segmented for all scored events with five seconds margins before and after each event. The study was approved by the Institutional Review Board of Gyeongsang National University Hospital (IRB No. 2014-02-010) and all experiments involving human subjects in this study were performed in accordance with the relevant regulations and guidelines. Written informed consents were obtained from all study participants.

### Pre-processing and image reconstruction

**Figure 1:**
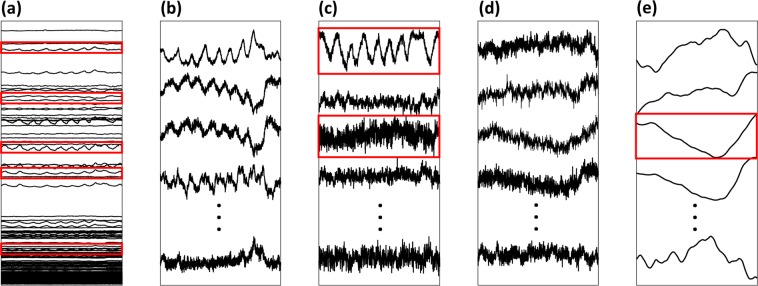
(**a**) Measured EIT data matrix of 208 × *N* where *N* is the number of scans in the chosen data segment. The selected data with high SNRs are shown in red boxes. (**b**) Enlarged views of the selected source signals with high SNRs. (**c**) ICA components where ventilation and blood flow artifacts, respectively are shown in red boxes. (**d**) Corrected source components. (**e**) Denoised source components where the third signal in the red box corresponds to the upper airway collapse during the obstructive apnea event.

For EIT imaging during sleep, sixteen electrodes were attached around the lower face as suggested by Kim *et al*.^14^. Currents were sequentially injected between each adjacent electrode pairs and induced voltages were measured between all adjacent electrode pairs to produce 16 × 16 = 256 data. For each current injection, three voltages data measured from two current-injection electrodes were excluded to remove the effects of the contact impedances. Therefore, one EIT scan produced 16 × 13 = 208 voltage data. The scan was repeated 50 times per second to be able to produce EIT images with a temporal resolution of 50 frames/s. The segmented EIT data corresponding to a respiratory event was expressed as 208 time series of scanned data in a form of 208 × *N* data matrix as shown in Fig. 1(a), where *N* is the number of scans during the respiratory event. From the acquired 208 times series, we chose sixteen of them having the highest signal-to-noise ratio (SNR) as shown in Fig. 1(b).

The measured EIT data from the lower face were influenced not only by upper airway occlusion but also by neck movements, blood flow in the carotid artery and respiratory motions (Fig. 1(c)). These artifacts were suppressed using the independent component analysis (ICA) proposed by Ayoub *et al*.^13^. Sixteen independent components were first produced as follows:

1 $${\bf{S}}={\bf{W}}{\bf{X}}$$

where **S** refers to the approximated independent source signals, **W** is the unmixing matrix and **X** is the 16 × *N* data matrix including the chosen EIT data segments with high SNRs. To remove the artifacts, we defined a modified unmixing matrix $\hat{{\bf{W}}}$ by replacing the columns of ${{\bf{W}}}^{-1}$ corresponding to the artifacts by zero columns. Then, we computed the corrected source components **U** as

2 $${\bf{U}}={\hat{{\bf{W}}}}^{-1}{\bf{S}}$$

where ${\hat{{\bf{W}}}}^{-1}$ is a modified mixing matrix and **U** is a 16 × *N* source matrix of corrected source components as shown in Fig. 1(d). The sixteen corrected source component signals were denoised with simple lowpass filters as shown in Fig. 1(e) and we should choose the component corresponding to upper airway occlusion such as component 3 for example.

**Figure 2:**
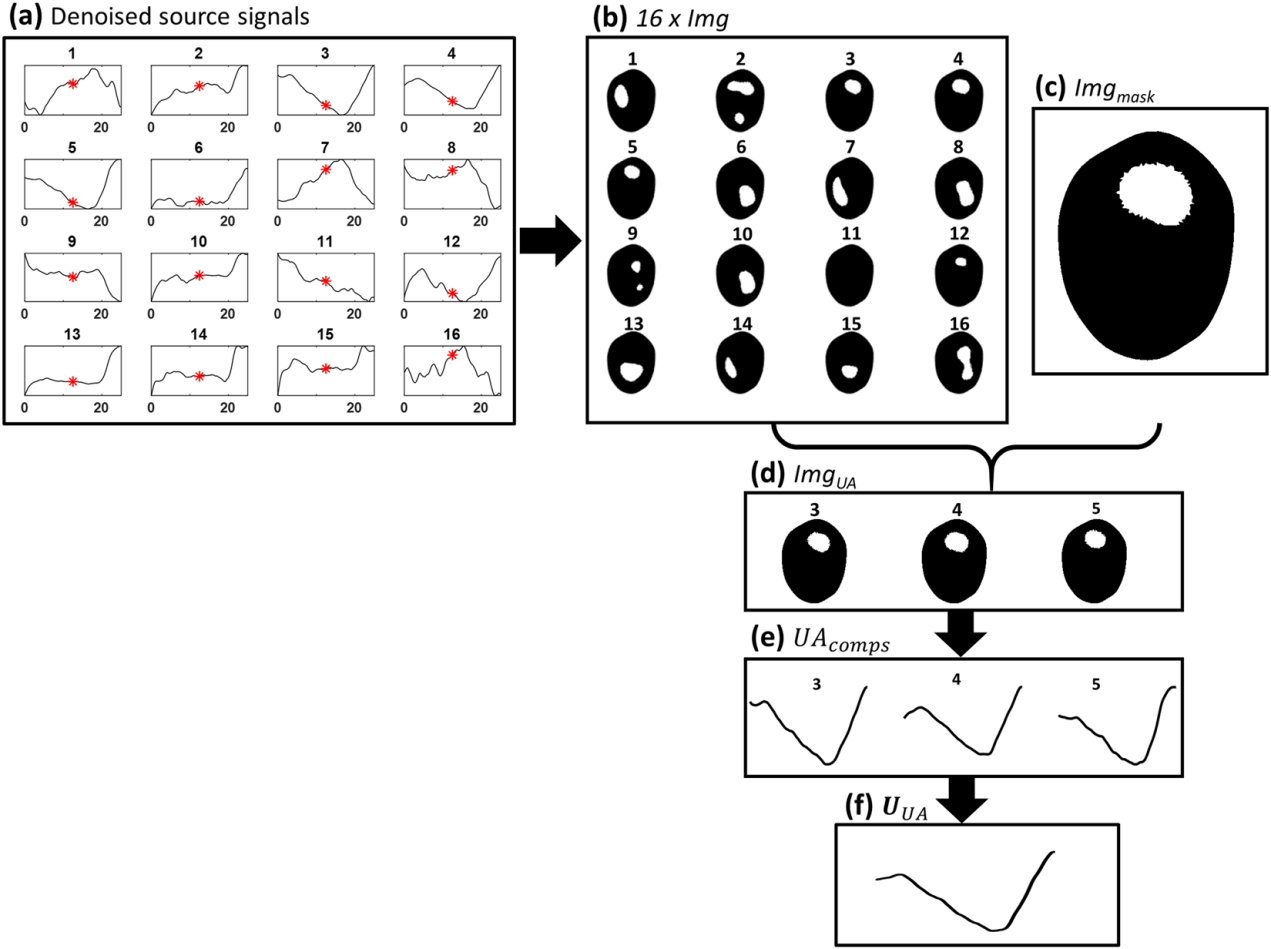
(**a**)Denoised source components, red asterisks refer to the midpoints of the components. (**b**) Reconstructed images (16 × *Img*) at the locations of red asterisks. (**c**) UA mask image (*Img*_*mask*_) from the swallowing maneuver. (**d**) Selected reconstructed images (*Img*_*UA*_) with high correlations with the UA mask image. (**e**) Selected UA signal components (***UA***_***comps***_). (**f**) Computed upper airway component (***U***_***UA***_).

In the previous work^13^, the source component corresponding to a time course of upper airway closure and reopening was manually selected from the denoised source matrix. In this paper, we replaced this manual step by adopting an image-based approach. We reconstructed sixteen sets of time-difference EIT images (16 × *Img*) using all sixteen denoised source components, respectively, at the midpoint of the event duration when the upper airway was closed as shown in Fig. 2(a,b). The reference EIT data for these time-difference EIT image reconstructions was selected during the normal breathing time when the upper airway was totally open. The image reconstructed by using a proper source component should clearly show the closed upper airway.

The upper airway mask image (*Img*_*mask*_) was calculated from the swallowing image using the threshold method. The threshold value was chosen as 40% of the maximum pixel values of the swallowing image as displayed in Fig. 2(c). The correlation between each reconstructed image *Img* and the upper airway mask image *Img*_*mask*_ was computed to select the images (*Img*_*UA*_) with a correlation factor greater than 75% as shown in Fig. 2(d). The components corresponding to the selected images were automatically chosen as the components correctly describing the upper airway dynamics during the event as displayed in Fig. 2(e). The upper airway component U_***UA***_ was computed as the average of the selected components corresponding to *Img*_*UA*_ (Fig. 2(f)). The time-series images were reconstructed, by fidelity-embedded regularization (FER) algorithm, after fitting U_***UA***_ to the original data size^18^. The conductivity change signal was computed as a sum of pixels then the upper airway closure signal (UA_closure) was expressed as the psercentage of conductivity changes.

### Feature extraction

**Figure 3:**
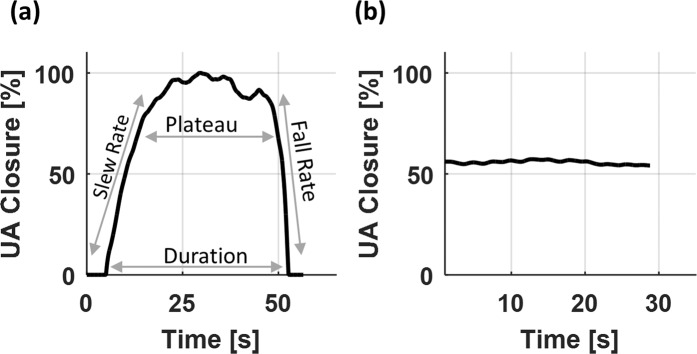
Two types of respiratory events: (**a**) C&O event and (**b**) C event.

According to the shape of UA_closure signal, we defined two types of events: C&O event and C event. In the C&O event, the upper airway starts closing, closes to a certain level and then reopens as shown in Fig. 3(a). On the other hand, in the C event, the upper airway remains partially closed as shown in Fig. 3(b). To express the behavior of the upper airway during the respiratory events, a set of features were defined from the UA_closure signal: maximum, duration, mean UA % closure, slew rate, fall rate, rise/fall ratio, plateau percent and fluctuation. The maximum (Max UA_closure) is the largest value of the UA_closure signal during the event. The duration is the time difference between the start of closing and the end of reopening. The mean UA % closure is a ratio between area and duration where area is calculated as the area under the UA_closure signal during the duration of the event. The slew rate is the upper airway occlusion speed from 10 to 90% of the maximum during the collapse phase. The fall rate is the upper airway reopening speed from 90 to 10% of the maximum during the reopening phase. The rise/fall is a ratio between the closing and opening times. Representing the time of sustained occlusion, the plateau time is defined as the time duration with more than 70% of the maximum closure during the event. The fluctuation is computed as the frequency of upper airway vibration during the event. Note that the slew rate, fall rate and rise/fall are obtained only from the C&O event.

### Statistical analysis

All data processing and statistical analyses were performed using MATLAB R2015a (Mathworks, Inc., Natick, Massachusetts, United States). We compared the AHI_EIT_ with the AHI_PSG_ by the Pearson correlation. A Bland-Altman plot was used to assess the agreement between EIT and PSG, where it graphically explains the difference between AHI_PSG_ and AHI_EIT_ against their average. The upper and lower limits of agreement were shown in the Bland-Altman plot with mean difference ± 1.96 SD, respectively, where SD is the standard deviation. The significant differences between event’s types were checked by the t-test with *p*-value < 0.05. The significant differences between patients’ features were determined by the one-way ANOVA Tukey test with p-value < 0.05. The patients’ features were displayed by box-and-whisker plots and heatmaps clustering the patients according to their features.

## Results

**Figure 4:**
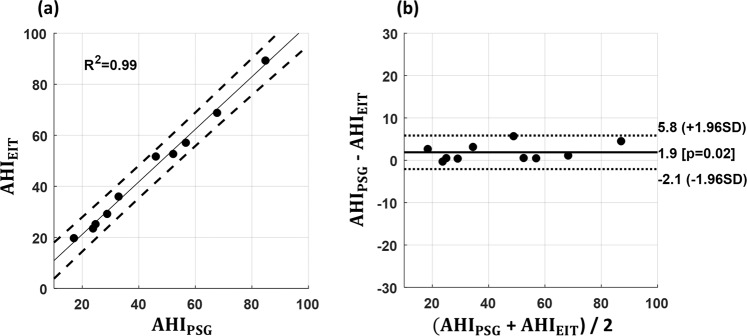
AHI comparison between EIT and PSG. (**a**) Linear regression plot where dashed lines indicate 95% confident intervals on both sides. (**b**) Bland-Altman plot where the solid line indicates the mean bias and dotted lines represent the limits of agreement.

Figure 4(a) shows a strong correlation between AHI_EIT_ and AHI_PSG_. The solid regression line was obtained with an estimated R^2^ value of 0.99 and two dashed lines refer to prediction bounds of 95%. The Bland-Altman plot in Fig. 4(b) comparing AHI_PSG_ and AHI_EIT_ shows the mean difference of 1.9 with limits of agreement ranging from −2.1 to 5.8. The upper airway EIT method slightly underestimated the AHI by the average of 1.9 events/h compared with the conventional PSG method.

**Figure 5:**
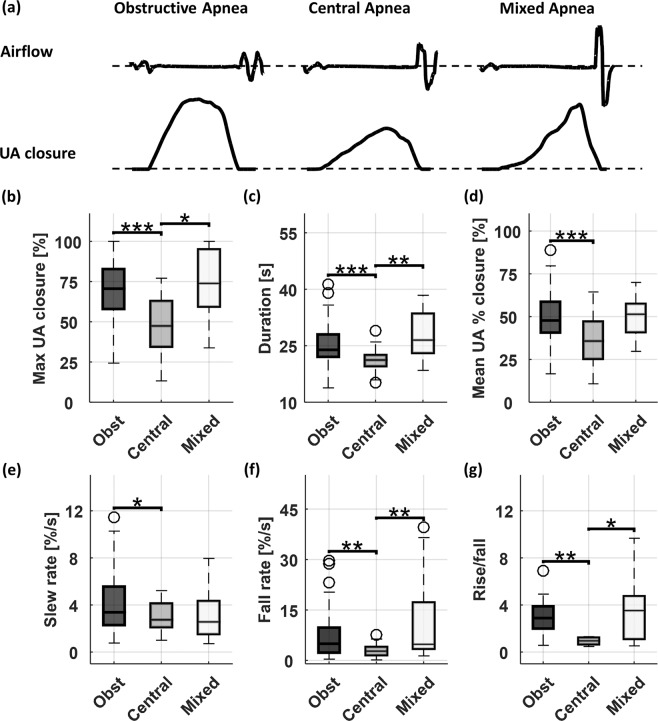
Upper airway dynamics of the first patient during different respiratory events including obstructive (Obst), central and mixed apneas. (**a**) The UA_closure signal from EIT and airflow signal from PSG during different events. (**b**–**g**) Comparisons of extracted features during obstructive, central and mixed apneic events. The black circles refer to the outliers. The asterisk symbols indicate the significant levels: *P < 0.05; **P < 0.01; ***P < 0.001.

Figure 5 shows the details of the upper airway dynamics of the OSA patient with 61 obstructive, 50 central and 19 mixed apneic events. The typical examples of those three types of events are shown in Fig. 5(a). The central apnea had a significantly lower degree of occlusion than obstructive and mixed apnea (Fig. 5(b)). The event duration was the shortest in central apnea. In addition, the area to duration ratio in central apnea was significantly lower than obstructive. Both central and mixed events showed slower occlusion compare with obstructive ones (Fig. 5(e)). In contrast, obstructive and mixed events exhibited faster reopening compared with central apneas (Fig. 5(f)). The rise/fall ratio was significantly lower in central apnea events compared with obstructive and mixed apneic events (Fig. 5(g)).

**Figure 6:**
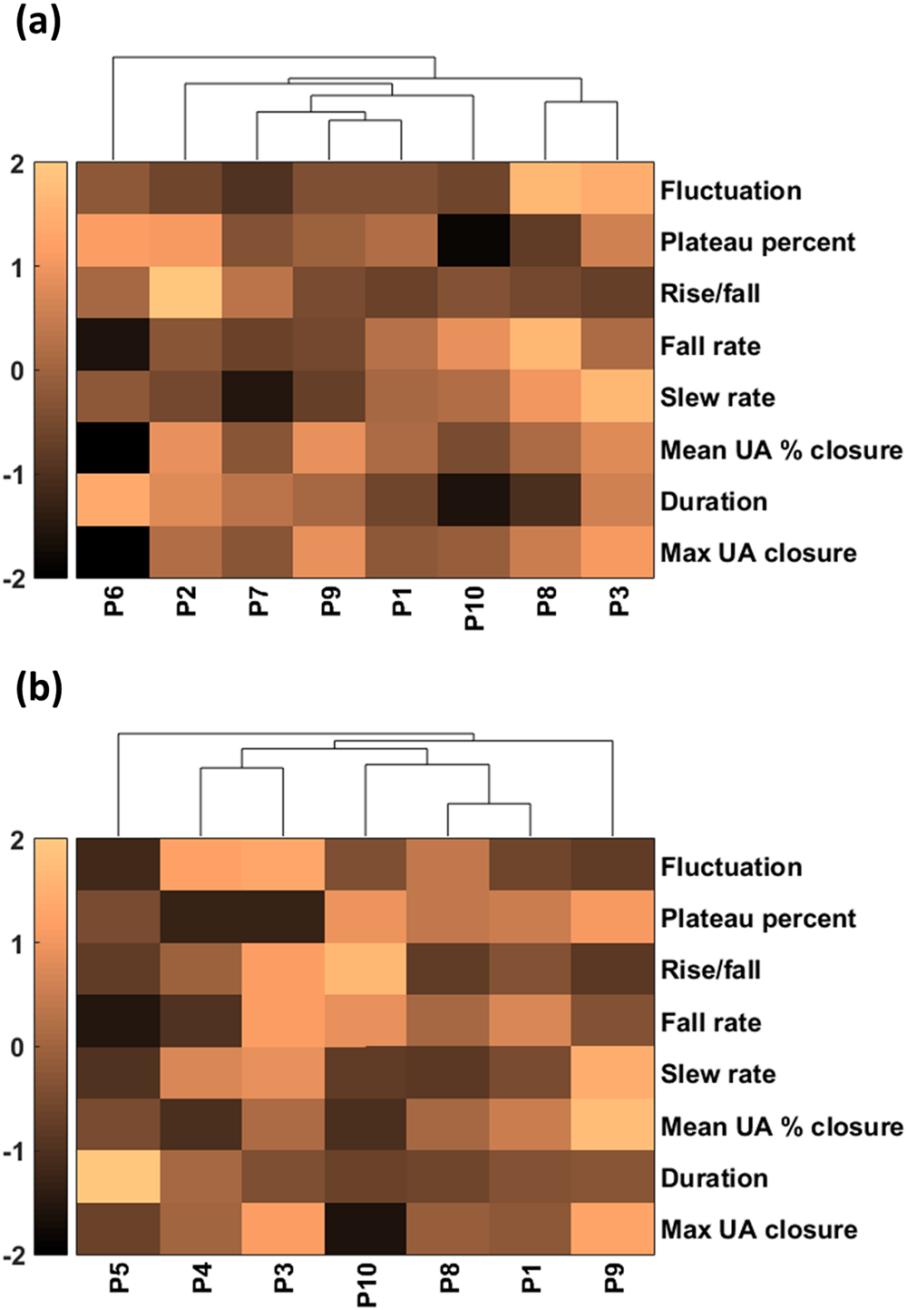
(**a**) Heatmap of the upper airway dynamics of 8 patients with obstructive apneic events. Each column corresponds to the individual patient. The computed hierarchical clustering showed that P1/P9 have similar behavior and P3/P8 belong to a different cluster. The behavior of P6 is different from all other patients. (**b**) Heatmap of the upper airway dynamics of 7 patients with hypopneic events. P1/P8 have similar behavior and P3/P4 belong to a different cluster. The behavior of P5 is different from all other patients.

**Figure 7:**
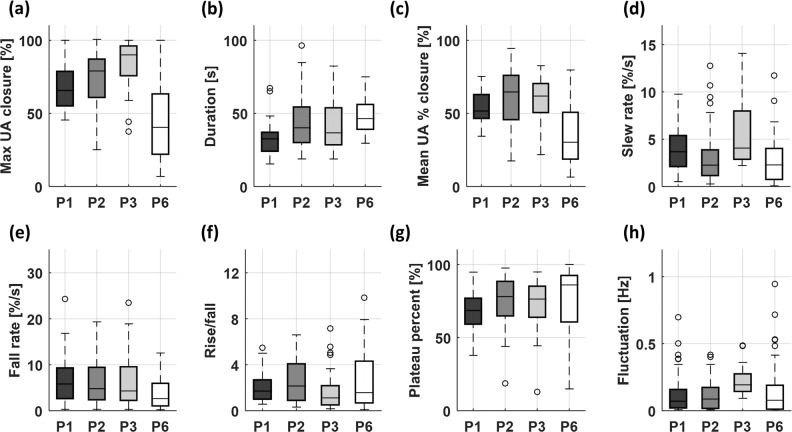
Comparison of features among four patients of P1, P2, P3 and P6. The open circles indicate the outliers.

Table 1 summarizes the features obtained from 8 patients with obstructive apneic events. The one-way ANOVA showed that all features were statistically significant among the patients except the rise/fall ratio. Table 2 presents a summary of features from 7 patients with obstructive hypopneic events and shows that the maximum, duration, mean UA % closure, fall rate, plateau percent, and fluctuation were significantly different among these patients. Figure 6 provides a graphical representation of the features using heatmaps. The heatmaps display the data in a grid view where each row represents a feature and each column represents a patient. The data in each raw was normalized to the same range [−2 2]. Figure 6(a) illustrates the features of 8 patients with obstructive apneic events where the patients were sorted based on their features. According to the hierarchal clustering method, both P3 and P8 belong to one class. P1 and P9 belong to another class and P6 is different from all others. In the same manner, the features of 7 patients with hypopneic events are shown in Fig. 6(b). Further analyses revealed the distribution of significant patients’ features, where one patient was chosen as a representative for each clustered class in the OSA heatmap. In Fig. 7, the features of four chosen patients (P1, P2, P3 and P6) were displayed by the boxplot.

**Table 1:** Comparison of features among 8 patients during obstructive sleep apneic events.

	P1	P2	P3	P6	P7	P8	P9	P10	*P* value
Max UA closure [%]	68.1	72.9	83.0	45.2	66.9	76.3	80.7	69.6	<0.001
Duration [s]	32.4	43.1	41.4	47.7	39.6	29.5	37.9	25.1	<0.001
Mean UA % closure [%]	53.9	60.3	59.4	35.1	50.5	54.0	60.5	48.9	<0.001
Slew rate [%/s]	3.9	3.1	5.9	3.5	1.8	5.1	2.9	4.1	<0.001
Fall rate [%/s]	6.8	5.9	6.6	3.8	5.3	8.9	5.5	7.8	0.011
Rise/fall	2.1	10.4	1.8	4.2	4.8	2.4	2.6	3.0	0.08
Plateau percent [%]	67.4	74.9	71.1	75.7	62.6	58.8	65.3	50.2	<0.001
Fluctuation [Hz]	0.12	0.11	0.22	0.13	0.09	0.23	0.12	0.11	<0.001

The numbers are mean values. The statistical significance was evaluated using the ANOVA test. The clinical significant needs to be tested with a larger number of patients.

**Table 2:** Comparison of features among 7 patients during hypopneic events.

	P1	P3	P4	P5	P8	P9	P10	*P* value
Max UA closure [%]	56.7	74.5	59.9	50.4	58.4	75.9	38.8	<0.001
Duration [s]	33.7	32.0	44.7	95.7	27.4	36.1	26.5	<0.001
Mean UA % closure [%]	48.9	45.2	33.2	39.0	44.3	61.7	33.2	<0.001
Slew rate [%/s]	3.2	4.8	4.6	2.6	2.8	5.4	2.9	0.64
Fall rate [%/s]	5.4	6.1	2.7	1.9	4.4	3.8	5.7	0.04
Rise/fall	1.7	3.0	2.0	1.3	1.3	1.3	3.4	0.82
Plateau percent [%]	69.9	52.7	52.9	60.4	68.7	74.8	73.7	<0.001
Fluctuation [Hz]	0.11	0.32	0.30	0.04	0.22	0.09	0.13	<0.001

The numbers are mean values. The statistical significance was evaluated using the ANOVA test. The clinical significant needs to be tested with a larger number of patients.

**Figure 8:**
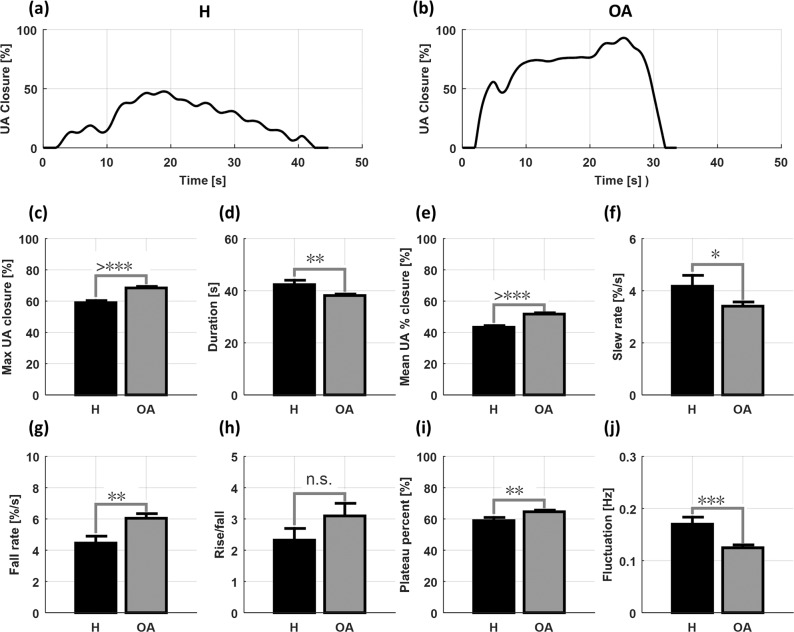
Upper airway dynamics of all 10 patients during apneic and hypopneic events. (**a**,**b**) Typical examples of the UA closure signals during hypopnea (H) and obstructive apnea (OA), respectively. (**c**–**j**) Features comparison between H and OA events using bar plots and standard errors. The black asterisk symbols indicate the significant levels and n.s. means non-significant: *P < 0.05; **P < 0.01; ***P < 0.001.

In Fig. 8, we elaborated the differences in all features between obstructive apneic and hypopneic events from all 10 patients. Figure 8(a,b) show typical examples of the UA closure signal during hypopnea (H) and obstructive apnea (OA), respectively. The maximum upper airway occlusion was higher during obstructive apnea than hypopnea (Fig. 8(c)). The duration was longer in the hypopneic events (Fig. 8(d)) and the mean UA % closure was larger in the apneic events (Fig. 8(e)). The slew rate was higher in the hypopneic events (Fig. 8 (f)) whereas the fall rate was higher in the apneic events (Fig. 8(g)). The plateau percent during apnea was greater (Fig. 8(i)) and the upper airway fluctuated more during the hypopneic events (Fig. 8(j)). However, the rise/fall ratio did not show a significant difference between obstructive apnea and hypopnea (Fig. 8(h)).

## Discussion

The purpose of this study was to provide additional information about the upper airway dynamics during respiratory events, which may help sleep clinicians to better understand the characteristics of the OSA patient and design an appropriate treatment plan. In order to automate the acquired EIT data for several hours of sleep, we first developed the automated data processing method to produce time-series EIT images of the upper airway during respiratory events. From the reconstructed EIT images, we could extract the UA closure signal expressing the upper airway dynamics during sleep apnea. Providing a multi-featured model that estimates upper airway occlusion patterns, we could demonstrate that the EIT technology can be used to characterize OSA patients based on their upper airway dynamics. Additionally, we could categorize obstructive apnea and hypopnea using the extracted features.

The Bland-Altman plot in Fig. 4 showed that the proposed EIT method slightly underestimated the AHI compared with the conventional PSG method. This could have stemmed from the limitation of the proposed method to detect respiratory events in the presence of an excessive amount of motion artifacts. On the other hand, this could be related with the choice of the correlation factor between *Img*_*mask*_ and *Img*. We chose the correlation factor heuristically in this paper based on the acquired data, but the chosen value of 75% could be suboptimal. If it was too high, we should have lost some UA information or signal. If it was too small, our method tend to add artifacts to the extracted UA signal. Future studies are needed to find an optimal correlation factor for a large dataset.

Currently, drug-induced sleep endoscopy (DISE) is considered to be a reliable method of upper airway evaluation. It provides three-dimensional dynamic visualization of the obstructive upper airway during a relatively short period of sedated sleep^19^. In contrast, the proposed EIT method is apt for long-term monitoring of the upper airway dynamics during natural sleep without sedating the patient. Since this study was restricted to two-dimensional EIT imaging of the upper airway, we suggest future studies of three-dimensional EIT imaging using at least two electrode layers.

Several attempts have been made to investigate the upper airway dynamics and breathing patterns during sleep^20,21^. The duration of respiratory events was suggested as a marker for the arousal threshold, where a low threshold is associated with a short duration^22^. Our results agree with these previous studies where the duration is shortest for central apnea and longest for mixed apnea^23^. On the other hand, our study showed that the degree of upper airway occlusion was significantly lower during central apnea. Moreover, a gradual occlusion of the upper airway was observed during central apnea. This finding is supported by the earlier study which showed that gradual progressive pharyngeal narrowing occurred during induced central apneic events^24^. Mixed apnea is a combination of central and obstructive apnea where central apnea is followed by obstructive apnea^17^. In this study, we found that the slew rate at the beginning of the mixed event is similar to that of central apnea, whereas its fall rate at the end of the mixed event is similar to that of obstructive ones.

Comparing the upper airway dynamics between obstructive apnea and hypopnea, we found that the upper airway behavior differs between apnea and hypopnea. The significantly larger amounts of upper airway closure during apnea events agree well with the definition of obstructive apnea and hypopnea^25,26^. The average duration was longer in hypopneic events compared with obstructive ones as described in the previous study by Muraja-Murro *et al*.^27^. In addition to this, our results showed that the plateau percent during apneic events is larger compared with hypopneic ones, whereas the fluctuation was larger in hypopneic events. The slew rate was higher in hypopneic events, but the fall rate was larger in apneic ones.

Since our results support the ability of the EIT method to distinguish between OA and H, we may develop new scoring criteria using the extracted features of the upper airway dynamics. We suggest using multiple features such as the mean UA % closure, max UA closure, fluctuation, duration, fall rate and slew rate though the mean UA % closure should be the primary feature. In our future study, we plan to adopt a machine learning technique with a multi-feature model to distinguish between OA and H using the extracted features. Future studies with more patients are needed to validate the possibility of the proposed EIT method to replace the conventional home sleep test or supplement existing in-lab PSG devices to improve the accuracy of the sleep apnea diagnosis.

## Conclusion

The proposed upper airway EIT imaging method can be applied to overnight sleep study to characterize the upper airway dynamics of respiratory events. Knowledge of common patterns of the upper airway dynamics during different types of respiratory events may lead to a new OSA phenotyping method for patient-specific treatment planning. Though we found significant differences in the extracted features among 10 OSA patients and different event types, the physiological meaning of these differences needs to be explained in future studies. Given the limitation of the available EIT device, we could conduct in this paper only the two-dimensional EIT imaging experiments of the lower neck. We suggest future studies of three-dimensional EIT imaging experiments including the retroglossal and retropalatal spaces for a large number of patients to provide more insights about the upper airway dynamics.
